# Diode Laser-Guided Protocol for Endo-Perio Lesions: Toward a Multi-Stage Therapeutic Strategy—A Case Series and Brief Literature Review

**DOI:** 10.3390/medicina61122157

**Published:** 2025-12-03

**Authors:** Ioana-Roxana Munteanu, George-Dumitru Constantin, Ruxandra-Elena Luca, Ioana Veja, Mariana-Ioana Miron

**Affiliations:** 1 University Clinic of Oral Rehabilitation and Dental Emergencies, Faculty of Dentistry, “Victor Babeș” University of Medicine and Pharmacy, Eftimie Murgu Square No. 2, 300041 Timișoara, Romania; munteanu.roxana@umft.ro (I.-R.M.); miron.mariana@umft.ro (M.-I.M.); 2 Interdisciplinary Research Center for Dental Medical Research, Lasers and Innovative Technologies, Revoluției 1989 Avenue No. 9, 300070 Timișoara, Romania; 3 Discipline of Clinical Practical Skills, Department I Nursing, Faculty of Medicine, “Victor Babeș” University of Medicine and Pharmacy, 300041 Timișoara, Romania; george.constantin@umft.ro; 4 Doctoral School, “Victor Babeș” University of Medicine and Pharmacy, Eftimie Murgu Square No. 2, 300041 Timișoara, Romania; 5 Department of Dental Medicine, Faculty of Dentistry, “Vasile Goldiș” Western University of Arad, 310025 Arad, Romania

**Keywords:** endo-perio lesion, diode laser, photobiomodulation, low-level laser therapy, periodontal regeneration, adjunct antimicrobial disinfection

## Abstract

*Background and Objectives*: This prospective case series evaluated a treatment strategy in endodontic-periodontal lesions resulting from concurrent pulpal and periodontal infections. These present significant management challenges, particularly when they exhibit resistance to standard treatment modalities. Persistent microbial biofilms in regions like dentinal tubules and lateral canals can make it hard for healing to happen, even with good endodontic and periodontal care. Diode lasers have antibacterial and photobiomodulatory effects, but they are most often used as single-stage disinfection techniques. This pilot study evaluated a multi-stage diode laser protocol designed to enhance healing outcomes in refractory endo-perio lesions that had not responded to conventional treatment. *Materials and Methods*: Twelve patients (aged 20–60 years) with chronic endo-perio lesions, referred after unsuccessful earlier treatment, were treated utilizing a sequential diode laser regimen: Phase 1—Endodontic disinfection: Following canal instrumentation (0.75 W, pulsed mode, frequency 15 Hz, 200 μm fiber, 15 J dosage/20 s) using a 976 nm diode laser. Phase 2—Periodontal disinfection: Following SRP, intra-pocket (0.75 W, pulsed mode, frequency 15 Hz, 300 μm fiber, 3.75 J dosage/5 s) using a 976 nm diode laser; Phase 3—Post treatment photobiomodulation: After periodontal and endodontic therapy, photobiomodulation was applied using a 650 nm diode laser intra-pocket and in the periapical region (25 mW, continuous mode, 1.5 J dosage) to reduce postoperative inflammation and stimulate healing. Clinical parameters—probing depth (PD), bleeding on probing (BOP), and mobility—along with radiographic bone fill were recorded at baseline and after 6 months. *Results*: All twelve cases showed measurable within-patient improvements over the six-month follow-up. Median probing depth decreased from 7.6 mm to 6.0 mm, and median bleeding on probing declined from 0.9 to 0.3. Radiographically, partial bone fill was observed in all cases, with a median value of 58.3 percent. Postoperative pain decreased progressively over the first 24 h, with patients reporting mild discomfort by 24 h. No adverse events were recorded. *Conclusions*: Within the limitations of this small, uncontrolled pilot study, the multi-stage diode laser protocol was associated with clinical and radiographic improvements and low postoperative discomfort in refractory endo-perio lesions. These preliminary findings suggest that such a protocol may serve as a useful adjunct to conventional therapy. Larger, controlled studies are required to confirm these outcomes and determine long-term efficacy.

## 1. Introduction

Endodontic–periodontal (endo-perio) lesions are pathological conditions that simultaneously affect the dental pulp and the periodontal supporting structures. These lesions often manifest as deep periodontal pockets associated with pulpal necrosis or infection, reflecting the close anatomical and functional interrelationship between the pulp and periodontium [1,2]. The presence of lateral and accessory canals, as well as the apical foramen, provides potential pathways for the spread of infection between the two tissues [3,4]. Because of this bidirectional communication, infection may originate from either the pulpal or periodontal component and subsequently involve the other. Consequently, even when one aspect of the lesion is treated successfully, residual microorganisms in the untreated component can compromise or delay overall healing [5,6]. Management of endo-perio lesions is particularly challenging. Conventional therapy typically involves root canal treatment for the endodontic component and scaling, root planing, or periodontal surgery for the periodontal component [7]. Adjunctive use of systemic antibiotics or antiseptic irrigants is often recommended in severe or refractory cases to enhance infection control [8,9]. However, treatment outcomes remain unpredictable, and persistent inflammation or incomplete healing is frequently reported [10].

A major limitation of standard mechanical and chemical disinfection methods is their inability to fully eradicate microbial biofilms located in complex anatomical areas such as dentinal tubules, lateral canals, and apical ramifications [11,12]. Furthermore, periodontal tissue regeneration and bone healing are often restricted, especially in chronic or combined lesions where both tissue types are significantly compromised [13,14]. These challenges highlight the need for advanced therapeutic modalities that can enhance decontamination and stimulate biological repair.

**Figure 1:**
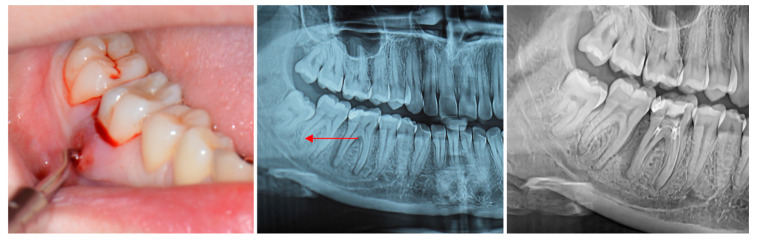
Clinical and radiographic findings of an endo-periodontal lesion of a lower first molar. The intraoral photograph (**left**) demonstrates localized gingival inflammation and bleeding upon probing in the molar region. The periapical radiograph (**center**) reveals baseline vertical alveolar bone loss adjacent to the affected tooth (indicated by the red arrow). The third radiographic view (**right**) shows advanced periodontal bone destruction post-treatment, evidence of failed conventional treatment.

Refractory endo-perio lesions refer to cases that fail to respond to conventional endodontic and periodontal therapy despite appropriate mechanical, chemical, and pharmacological treatment (Figure 1) [15]. These lesions persist or recur due to complex microbial or anatomical challenges, or host-related factors that limit healing [16,17]. In such situations, persistent infection in dentinal tubules, accessory canals, or apical deltas can act as bacterial reservoirs, leading to reinfection or delayed resolution [18]. Bacteria can persist through several processes, including biofilm formation, penetration and retention inside dentinal tubules, survival in nutrient-deficient settings, possession of virulence factors that modify host responses, and resistance to antimicrobial treatments [19].

The term refractory implies that the lesion remains resistant to standard therapy, either from the outset (primary resistance) or after an initial but incomplete healing response (acquired resistance). To accomplish this objective, endodontic therapy has depended on a chemo-mechanical debridement of the root canal system [20]. About 30–45% of the root canal system is still untouched by mechanical instruments since the architecture of the root canal is so complicated. Excessive instrumentation may further compromise the tooth’s integrity or even induce an apical fracture [21]. Consequently, there is increased reliance on the effectiveness of disinfectants in eradicating biofilm bacteria rather than planktonic bacteria. Biofilm bacteria can be up to 1000 times more resistant to antibacterial agents than planktonic bacteria [22]. These cases are particularly difficult because even when the pulpal infection is eliminated, the periodontal component may continue to harbor pathogenic biofilms within cemental irregularities and deep osseous defects [23].

Host immune modulation and systemic factors like diabetes, smoking, or not being able to heal properly can also make recovery even harder [24]. So, endo-perio sores that don’t heal on their own often need more advanced or extra treatments besides just mechanical and chemical disinfection [25]. These extra methods can get rid of bacteria more efficiently and help the body heal itself [26]. In recent years, laser-assisted therapies have gained popularity in dentistry as supplementary instruments that facilitate dental treatment and accelerate wound healing [27]. Diode lasers (wavelength 810–980 nm) are distinguished among available laser systems for their efficacy in targeting pigmented tissues and their ability to penetrate deep into soft tissues and root dentin [28]. When diode lasers are set to higher powers, they have photothermal effects that break down bacterial cell walls and biofilms. This makes chemical watering methods more effective. Nearly all of the laser light can pass through the root dentin around it because the wavelength of 445 nm is good at being absorbed [29]. The coloured components of the bacteria absorb a significant amount of laser energy, which is then released locally as heat. When the temperature rises, the germs die. This means that an antibacterial effect can be found even in the deep tissue layers and at the base of the dentinal tubules [30]. Laser light may also be able to reach the apical inflammatory processes in that area, where the dentin isn’t very thick [31].

At lower power outputs, diode lasers induce photobiomodulation (PBM), also known as low-level laser therapy (LLLT), which exerts anti-inflammatory and biostimulatory effects by stimulating mitochondrial activity, enhancing ATP production, and modulating growth factor release [32]. These effects accelerate tissue repair processes in both the pulp and periodontal structures, contributing to improved healing dynamics. Several clinical studies have highlighted the dual benefits of diode laser use in endodontics and periodontics. For example, the addition of a 940 nm diode laser to conventional endo-perio therapy significantly reduced probing depths, tooth mobility, and radiographic bone loss over six months [33]. Similarly, the 980 nm diode laser used post-obturation has been shown to markedly decrease postoperative pain compared to conventional therapy [34]. In another study, Low-level laser therapy (LLLT) utilizing diode lasers demonstrated enhanced management of postoperative pain and swelling in comparison to traditional irrigation methods and laser-activated irrigant agitation [35].

Collectively, these findings suggest that diode lasers can serve a dual role—providing microbial decontamination through photothermal effects and biological modulation through photobiostimulation—thereby improving both clinical and patient-centered outcomes [36]. However, most existing applications are limited to single-stage adjunctive use, focusing only on either disinfection or postoperative pain reduction. There remains a significant gap in the literature regarding systematic, multi-phase laser protocols aligned with sequential stages of wound healing—namely, infection control, inflammation modulation, and tissue regeneration [37].

We acknowledge that the EFP S3 guideline does not suggest diode lasers as routine adjuncts due to heterogeneous evidence from studies in untreated periodontitis. However, these recommendations do not address refractory endo-perio lesions. Our previous work and consistent clinical outcomes in such cases provided the rationale to explore this multi-stage diode laser protocol as a feasibility and safety–focused case series.

## 2. Materials and Methods

This study was designed as a prospective case series including twelve teeth with refractory endo perio lesions, and was conducted to evaluate the clinical and radiographic effects of a multi-stage diode laser protocol in managing these cases. The study included 12 patients (5 males and 7 females) aged 20–60 years, all of whom presented with persistent endo-perio lesions that had failed to respond to conventional endodontic and periodontal therapy performed previously by other clinicians, and had a clinical presentation compatible with an endodontic-periodontal pathway of disease progression. Patients included in the study had already undergone standard endodontic and periodontal treatment, with persistent deep periodontal pockets (>6 mm) and radiographic bone loss for at least three months prior to referral.

Inclusion criteria were:

- Presence of a deep periodontal pocket (>6 mm) adjacent to a necrotic or previously treated tooth.
- Radiographic evidence of periapical and/or periodontal bone loss.
- No response to previous standard endodontic and periodontal treatments for at least three months.
- Good systemic health and willingness to comply with follow-up visits.

Exclusion criteria included:

- Teeth with vertical root fractures, advanced mobility (Grade III), or non-restorable crowns.
- Patients with uncontrolled systemic diseases, pregnancy, smoking, or recent antibiotic or anti-inflammatory therapy.

Ethical Considerations

The study was conducted in accordance with the Declaration of Helsinki [38] and was approved by the Ethics Committee of the ‘Victor Babeș’ University of Medicine and Pharmacy in Timișoara (Comisia de Etică a Universității de Medicină și Farmacie ‘Victor Babeș’ din Timișoara): approval code: no. 88/03.05.2024 rev 2025, approval date: 03 May 2024. All patients provided written informed consent before participation.

Treatment Protocol

Patients first received standard non-surgical endodontic therapy following accepted clinical protocols [39,40]. After isolation with a rubber dam (KerrHawe SA, Bioggio, Switzerland), root canals were instrumented using hand K-files and rotary NiTi files (FANTA DENTA, Shanghai, China) in a crown-down technique [41]. Irrigation was performed with sterile distilled water and 2.5% sodium hypochlorite (NaOCl) (CHLORAXID, CERKAMED, Stalowa Wola, Poland) during instrumentation to ensure effective debridement and microbial reduction. After instrumentation, the canals were dried out and an intracanal medication of calcium hydroxide (Calcipast, CERKAMED, Stalowa Wola, Poland) was used for one week to improve disinfection [42]. All patients had previously received standard non-surgical periodontal therapy (scaling and root planing, oral hygiene reinforcement, and infection control measures) at least six weeks before enrollment in this study to ensure resolution of acute inflammation and to confirm the refractory nature of the lesions [43]. Nonsurgical periodontal therapy is preferable for managing gum disease. Reduction in inflammation, pocket depth, and clinical attachment formation is achievable. The specific initial probing pocket depth at which nonsurgical periodontal treatment ceases to be effective remains unidentified. However, root monitoring should only be performed on 4 mm or deeper sites. This is because instrumenting shallow sites may cause attachment loss. Scaling and root planing may be performed as frequently as clinically necessary, similar to other periodontal interventions, to maintain optimal periodontal health. The dentist and patient must work together for periodontal treatment to succeed. Therefore, a good root debridement and risk factor modification approach (including good oral hygiene, patient education and motivation, stopping smoking, controlling diabetes, and healthy lifestyle modifications) are crucial to treating periodontitis [44].

The development of multi-wavelength diode laser technology significantly enhanced the therapeutic efficacy of laser treatments. These advanced modules can simultaneously target multiple tissue types and depths by integrating several wavelengths within a single device. This adaptability facilitates broader and more efficient treatments across various applications. Diode laser manufacturers lead in technological advancement, providing innovative solutions that integrate two or three different wavelength emitters within a single module. Multi-function laser modules offer superior power stability and beam quality, rendering them suitable for aesthetic medicine, laser surgery, and various therapeutic applications [45].

All patients underwent standardized multi-stage diode laser therapy using three different wavelengths corresponding to specific treatment phases:

Phase 1—Endodontic disinfection: Following access preparation and canal instrumentation, intracanal (0.75 W, pulsed mode, frequency 15 Hz, 200 μm fiber, 15 J dosage/20 s) was performed using a 976 nm diode laser. The laser fiber was moved circumferentially within the canal for 20 s per site.

Phase 2—Periodontal disinfection: Following SRP, intra-pocket (0.75 W, pulsed mode, frequency 15 Hz, 300 μm fiber, 3.75 J dosage/5 s) was performed using a 976 nm diode laser. The laser fiber was moved slowly, sweeping or circular motion along the root surface for 5 s per site.

Phase 3—Post treatment photobiomodulation: After periodontal and endodontic therapy, photobiomodulation was applied using a 650 nm diode laser (100 mW, pulsed mode) intra-pocket for 30 s to promote soft tissue regeneration and bone remodeling and in the periapical region (25 mW, continuous mode, 1.5 J dosage) for 60 s to reduce postoperative inflammation and stimulate periapical healing.

All laser applications were performed using a multi-wavelength diode laser device—Woodpecker^®^ Laser Blue (Guilin Woodpecker Medical Instrument Co., Guilin, China), which provides 450 nm, 650 nm, and 976 nm outputs in a single unit. Appropriate safety measures, including the use of wavelength-specific protective goggles, were strictly observed [46].

Each wavelength corresponds to a distinct biological target—microbial reduction, inflammation modulation, and tissue repair. A minimum one-week interval was maintained between phases and reassessment, to distinguish the biological effect of laser protocol. The schematic summarizes the rationale of the sequential photobiomodulation approach, integrating antimicrobial efficacy with regenerative stimulation for enhanced healing in refractory endo-perio lesions.

**Figure 2:**
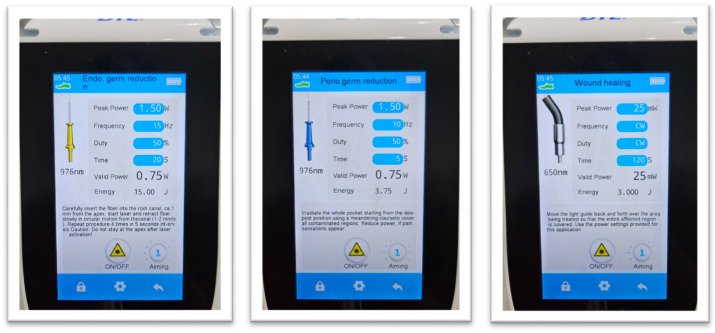
Laser device settings used for different phases of our protocol: endodontic germ reduction, periodontal germ reduction and photobiomodulation.

Although the device includes a 450 nm blue diode, this wavelength was not used in the clinical protocol; only the 976 nm (disinfection) and 650 nm (photobiomodulation) wavelengths were applied (Figure 2). Laser parameters for each treatment phase were selected based on existing literature and laser settings to balance antimicrobial efficacy and photobiomodulatory benefits while avoiding thermal damage to peri-radicular tissues [47,48]. Phases 1, 2 and 3 were carried out under aseptic conditions by a single experienced operator, with expertise in the use of laser therapy in dentistry, to ensure protocol consistency, and single visit [49].

Data collection

Clinical and radiological assessments were performed at baseline and 6 months post-treatment. The following parameters were recorded:

○ Probing depth (PD)
○ Clinical attachment level (CAL)
○ Tooth mobility (Miller’s index)
○ Postoperative pain using a visual analog scale (VAS)
○ Radiographic bone fill

Digital radiographs were used to assess bone healing, with baseline and follow-up images obtained using identical angulation, exposure settings, and positioning devices to ensure reproducibility [50]. Bone fill percentage was quantified, enabling objective and standardized measurement of radiographic bone regeneration while minimizing observer variability [51]. This approach provided consistent, reproducible quantification of periapical and periodontal bone changes between baseline and follow-up images, enhancing the accuracy of radiographic outcome assessment.

Clinical Evaluations

Periodontal charting documents essential clinical information on the gingiva, periodontal ligament, and alveolar bone to evaluate periodontal health. This diagnostic tool assesses probing depths, clinical attachment levels, bleeding on probing, recession, furcation involvement, mobility, and additional parameters. The goal of periodontal charting is to assess periodontal health, identify early disease markers, follow disease development, and plan therapy. Clinicians can diagnose gingivitis and periodontitis, assess intervention efficacy, and customize periodontal care for each patient.

**Figure 3:**
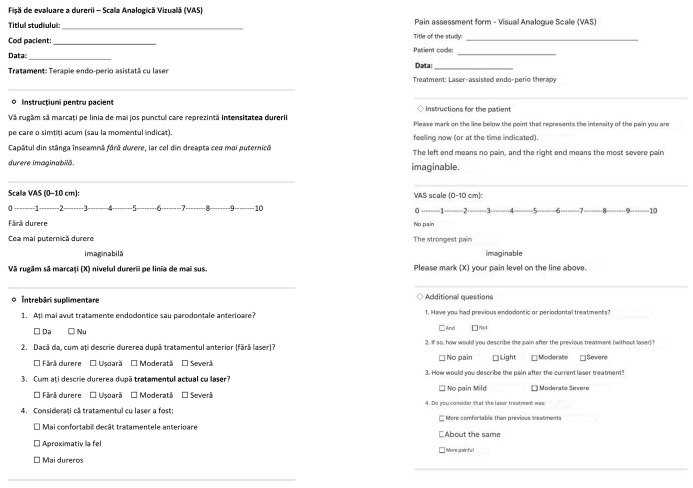
Example of a Visual Analog Scale (VAS) form used for pain assessment after laser-assisted endo-perio treatment. The form includes patient instructions, a 0–10 pain scale, comparative questions regarding previous and current laser treatments, and a section for recording pain scores at various postoperative time intervals. On the right the translated version.

Clinical measurements were recorded at baseline (pre-treatment) and 6 months post-treatment by a calibrated examiner to ensure consistency and reproducibility [52]. Probing depth (PD) was recorded at six sites per tooth and clinical attachment level (CAL) was measured using a manual periodontal probe (Table 1). The deepest pocket depth on the affected tooth was used for analysis [53]. Endodontic-periodontal lesions were first categorized according to the terminology recommended by the World Workshop for Classification of Periodontal Diseases (1999) [54], which distinguishes between: (1) endodontic-periodontal lesions, (2) periodontal-endodontic lesions, and (3) combined lesions. Based on these foundational categories, the most widely used classification of endodontic-periodontal lesions is the system proposed by Simon et al. [55], which stratifies lesions according to the primary etiology into five types (I–V). In the present case series, all included lesions corresponded to Type III (primary endodontic disease with secondary periodontal involvement). Tooth mobility was graded I–III according to Miller’s index [56]. Patient-reported pain was assessed using a 10-step Visual Analog Scale (VAS; 0 = no pain, 10 = worst pain). As this is a case series without a control group, VAS scores were analysed within each patient only, describing changes in pain intensity immediately after treatment, at 6 h, and at 24 h. The questionnaire also included an item asking patients to recall discomfort experienced after previous conventional (non-laser) endodontic or periodontal treatments; this information was used solely as a self-reported historical reference for each individual, not for inter-group comparison. No statistical comparison between laser therapy and conventional therapy was performed, as no control group was included (Figure 3) [57].

Radiographic assessment: Standardized periapical radiographs were obtained at baseline and 6 months using similar exposure parameters. The percentage of bone fill in periapical and vertical osseous defects was calculated by comparing baseline and follow-up radiographs to measure the filled area within the original defect outline. Radiographic bone healing was also categorized as complete, partial, or none based on visual interpretation [58].

## 3. Data Statistical Analysis

Given the case-series design and small sample size, statistical analysis was limited to descriptive methods. Continuous variables are reported as median values with interquartile ranges (IQR). No inferential statistical tests, effect sizes, or confidence intervals were used, as these would imply analytic comparisons not supported by the study design. Because this was an exploratory case series, no sample size calculation or power analysis was performed. Radiographic bone fill was quantified using a standardized polygon region of interest (ROI) on the affected site apical to the CEJ, expressed as the relative reduction in radiolucent pixels and corroborated by mean gray-level increase. Image analysis was performed using ImageJ v1.53 (developed and maintained by the National Institutes of Health (NIH), Bethesda, MD, USA). It is an open-source image processing package that includes preinstalled plugins useful for biomedical image analysis. No multiplicity adjustment or imputation was applied; analyses were performed in IBM SPSS Statistics **30.0.0**. Given the small sample size (*n* = 12) and absence of a control group, the study was exploratory and descriptive in nature. The inferential results and effect size estimates are presented to illustrate potential trends and should be interpreted with caution.

## 4. Results

**Figure 4:**
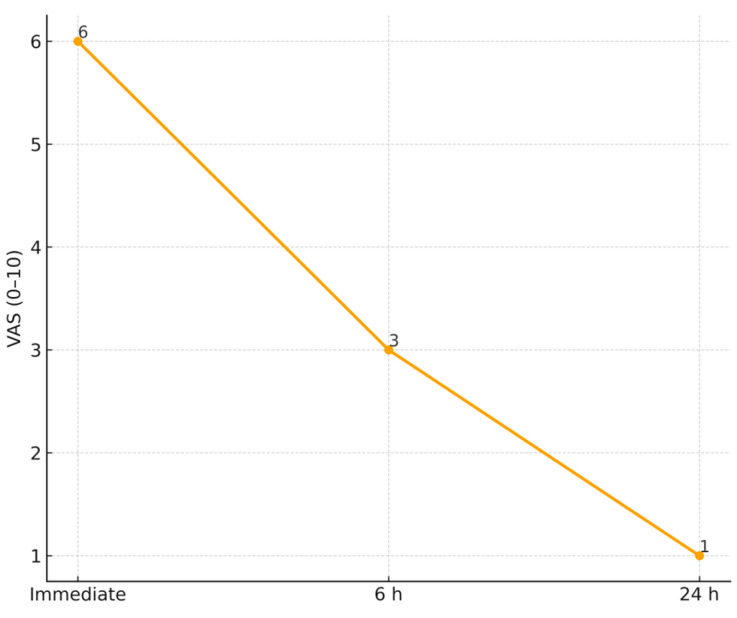
Visual Analog Scale (VAS) pain scores recorded immediately after treatment, at 6 h, and at 24 h. Values represent median within-patient pain levels for the 12 treated endo-perio lesions. A progressive decrease in discomfort was observed over the first 24 h, with pain shifting from moderate immediately post-treatment to mild levels by 24 h. No inferential comparisons were performed; data are presented descriptively in accordance with the case-series design.

Twelve endo-perio lesions classified as Type III according to Simon et al. [55] were included. Descriptive analysis showed within-patient reductions in probing depth, bleeding on probing, pain intensity, and radiographic lesion size after six months. Median probing depth decreased from 7.6 mm at baseline to 6.0 mm at six months (median change −1.5 mm, IQR −2.0 to −1.0). Median bleeding on probing declined from 0.9 to 0.3 (median change −0.6, IQR −0.8 to −0.4). Radiographically, the median percentage of bone fill at six months was 58.3 percent (IQR 50.0–66.7 percent). Pain intensity decreased within patients over the first 24 h, shifting from moderate–severe immediately after treatment to mild levels by 24 h (Figure 4). No inferential comparisons were made.

**Figure 5:**
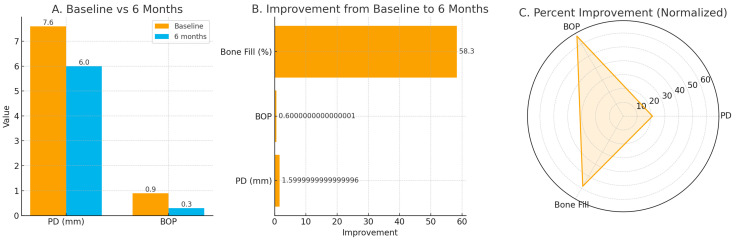
Composite panel illustrating clinical, radiographic, and patient-reported outcomes from baseline to 6 months in this case series of endodontic-periodontal lesions (*n* = 12). (**A**) Baseline vs. 6-month comparison of probing depth (PD) and bleeding on probing (BOP), presented as descriptive values using the study medians. (**B**) Horizontal improvement plot showing within-patient changes: PD reduction (−1.6 mm), BOP reduction (−0.6), and radiographic bone fill (+58.3 percent), displayed as positive improvement values. (**C**) Normalized spidergram illustrating relative percent improvements across PD, BOP, and bone fill.

Bleeding on probing showed a clear within-patient reduction over the 6-month period, decreasing from a mean of 0.9 ± 0.3 at baseline to 0.3 ± 0.5 at 6 months, indicating improved soft-tissue response and reduced inflammatory signs. Radiographically, the percentage of bone fill increased from a baseline mean of 29.2 percent to 58.3 percent at 6 months, reflecting partial osseous repair within the treated defects. AI-assisted image analysis supported these observations by demonstrating increased radiodensity and mineral deposition in most lesions, although complete defect resolution was not observed and residual radiolucency remained in several sites. Overall, the descriptive findings indicate progressive healing in refractory endo-perio lesions (Figure 5) following the multi-stage diode-laser protocol; however, as this is a case series with a small sample (*n* = 12) and no control group, these observations should be interpreted cautiously and viewed as exploratory.

### Radiographic Evaluation

Digital images were imported into ImageJ (v1.53), and calibration was performed using a known reference length (the measured canal length), establishing a pixel-to-millimeter conversion factor for subsequent measurements. A rectangular region of interest (ROI) was placed over the apical radiolucency, defined by the borders of the lesion at baseline and replicated at the six-month follow-up using the ROI Manager tool. Bone fill was quantified by comparing the radiolucent area (mm^2^) between time points.

At baseline, a distinct periapical radiolucency was evident around the root apex, extending along the mesial root surface, consistent with a deep periodontal pocket and loss of alveolar support.

**Figure 6:**
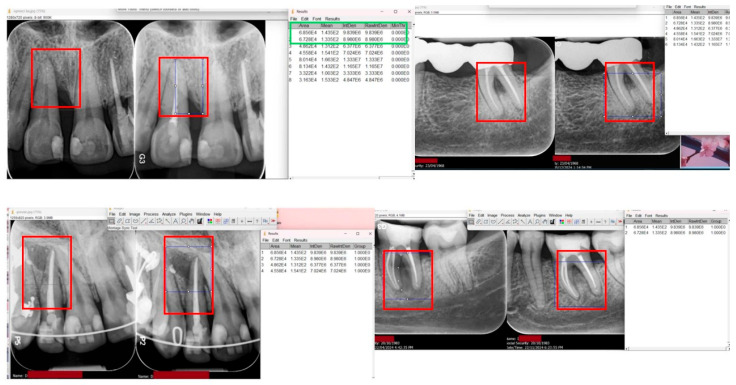

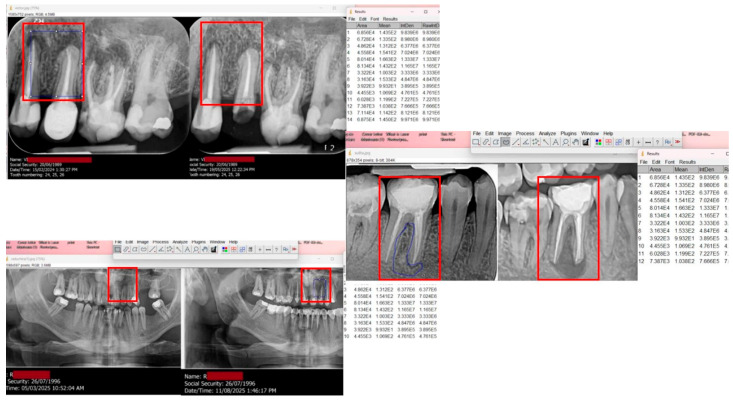
Periapical radiographs of maxillary anterior teeth showing bone density measurements using ImageJ software. The **left panel** shows the baseline radiograph, and the **right panel** displays the follow-up image after treatment. The rectangular region of interest (ROI) on the right image indicates the area selected for grey-scale density analysis. Quantitative results, including area, mean grey value, and integrated density, are presented in the Results window to the right.

At 6-month follow-up, the radiolucent area showed a marked reduction in size and density, with partial trabecular reformation and reappearance of the lamina dura around the apex and mesial surface (Figure 6).

These changes indicate approximately 40–60% bone fill and stabilization of the periodontal defect, reflecting a favorable, though incomplete, regenerative response following laser-assisted therapy.

## 5. Discussion

This pilot study provides preliminary evidence that a photobiomodulation-guided multi-stage diode laser protocol can substantially improve the management of combined endodontic-periodontal lesions. By sequentially targeting the key challenges at each phase of healing—infection control, inflammation/pain, and tissue regeneration—the laser-assisted strategy produced better clinical outcomes than conventional therapy alone [59,60]. Notably, our findings showed deeper pocket closure, greater attachment gain, more bone fill, and less postoperative pain. These improvements underscore the potential of moving diode lasers from a one-dimensional disinfection adjunct to a multi-phase therapeutic tool. One notable outcome was the reduction in probing depths and the marked improvement in bone fill observed with the use of the diode laser, indicating the effectiveness of the laser protocol in promoting periodontal healing [61]. The incorporation of photobiomodulation enhances periodontal healing around the damaged tooth, as evidenced by research highlighting its positive effects on tissue repair and inflammatory modulation [62]. From a biological perspective, photobiomodulation may have contributed to the observed soft-tissue improvement and partial bone fill by influencing several molecular pathways. Red and near-infrared irradiation can upregulate osteogenic mediators such as BMP-2 and VEGF, enhance mitochondrial activity with increased ATP production, and modulate inflammatory cytokines including IL-1β and TNF-α, creating a microenvironment more favorable for tissue repair. Although these mechanisms were not directly assessed in our study, they offer a biologically plausible explanation for the healing patterns observed in this case series. Photobiomodulation (PBM) in Phase 3 may have biostimulated periodontal ligament cells and osteoblasts, thereby enhancing the repair of the attachment apparatus [63]. Our findings align with those of Dembowska et al. (2022) [33], who similarly observed enhanced PD reduction and reduced mobility with the incorporation of a 940 nm diode laser in endo-perio treatment. Laser energy has the potential to decontaminate regions such as lateral canals or deep pocket niches that conventional methods find challenging to access, thus fostering a more conducive environment for reattachment [64]. Furthermore, the modulation of the inflammatory response by PBM likely facilitated a more organized healing process of the periodontal tissues, characterized by reduced chronic inflammation and enhanced regeneration [65]. When laser light interacts with biological tissues, photons are absorbed by chromophores such as melanin, haemoglobin, and water, converting optical energy into thermal or mechanical effects that produce clinical outcomes like bacterial destruction, coagulation, and vaporization. Among all laser–tissue interactions, absorption is the most critical, as it governs energy transfer and the therapeutic effect. Near-infrared (NIR) diode lasers (800–980 nm) are mainly absorbed by melanin and haemoglobin, showing high transmission through water and strong bactericidal effects against pigmented endodontic pathogens. Although 810 and 940 nm lasers offer deeper dentinal penetration due to lower water absorption, the 970–980 nm wavelength provides distinct advantages for endodontic and periodontal disinfection. Its slightly higher water absorption coefficient (μa ≈ 0.25 cm^−1^) promotes efficient localized thermal effects, inducing bacterial cell wall disruption, biofilm destabilization, and microcavitation that enhance irrigant activation and cleaning of endodontic and periodontal structures.

Importantly, the laser power settings and energy doses (expressed in Joules) were carefully calibrated to remain above the threshold required for effective bacterial and biofilm elimination but below levels that could induce thermal damage to dentin or periodontal tissues. These settings were chosen to provide sufficient fluence for bactericidal efficacy while maintaining surface temperatures below the critical threshold for thermal injury (<7–10 °C increase) [66].

The dramatic difference in postoperative pain between groups is in line with previous studies highlighting the analgesic effect of diode lasers. Kaplan et al. (2021) [34] found that patients who received a 980 nm laser application after RCT experienced significantly less pain in the first 48 h, which aligns with our observation of lower VAS scores after laser treatment. The mechanism is thought to involve laser-induced PBM by reducing the release of inflammatory mediators and improving local microcirculation, thereby accelerating the resolution of inflammation [67]. Similarly, Ismail et al. (2023) [35] demonstrated that PBM was more effective than conventional irrigation or even laser-activated irrigation in controlling post-endodontic pain [68]. By using the 810 nm laser transcutaneously on the apical area (Phase 2), we likely harnessed this effect, leading to quicker pain relief and possibly a reduced need for analgesics in the laser group—an important patient-centered benefit.

Radiographic outcomes from our pilot study also suggest a possible regenerative advantage with the laser protocol. By 6 months, laser-treated lesions showed approximately double the bone fill percentage of controls, acknowledging that 6 months is an early endpoint for definitive bone regeneration [69]. Two-dimensional periapical radiographs were employed to assess the efficacy of bone healing. This approach complicates the precise assessment of three-dimensional regeneration. Two-dimensional imaging is incapable of assessing the dimensions of buccolingual abnormalities, the thickness of the cortical plate, or volumetric alterations. It is susceptible to projection errors, even with the angle accurately adjusted. Therefore, the documented extent of radiographic bone fill in this case series should be interpreted cautiously, as it may either underrepresent or exaggerate the true regenerative response. The case series by Pedraza et al. (2025) provides supporting evidence that diode lasers can promote periapical healing; in their study, laser-disinfected root canals achieved favorable tomographic healing in a majority of primary endodontic lesions [70]. The authors attributed this to the laser’s ability to sterilize beyond the reach of irrigants and stimulate healing responses in periapical tissues [71]. Researchers demonstrated the impact of red and infrared light on essential biological pathways through interactions with certain photoacceptors within the cell, notably in mitochondria. Low-level laser therapy, also known as PBM, is a new medical term that describes how light interacts with cells to change their metabolism by increasing oxygen use and ATP synthesis through mitochondria. Our multi-stage approach builds on that concept by also addressing periodontal bone defects via LLLT (Phase 3), which may upregulate growth factors and enhance osteoblastic activity [72]. In addition to the clinical results, it is important to consider how this laser-guided method compares with more traditional approaches, especially the usage of systemic antibiotics. The diode laser protocol offers a localized antimicrobial effect without systemic exposure and adds biostimulation—benefits antibiotics do not provide. Safety considerations for intraoral lasers are well documented when standard precautions are followed [73]. Advantages of diode lasers over antibiotics in this context include localized antimicrobial action, no systemic side effects, no contribution to resistance, and added biostimulatory effects [74]. So, adding laser therapy to endodontic-periodontal treatments could help cut down on the need for antibiotics and give both antimicrobial and regenerative benefits when used correctly. Instrumentation by itself remains insufficient for the treatment of periodontitis. Manual instrumentation, deprived of the advantages of laser therapy, might end up in the accumulation of bacteria and debris inside the periodontal pocket and on the root surface. The pathogens remaining in the gingival tissue proliferate and persist in harming the periodontium. This could lead to a faster return of periodontal problems, such as bone loss, tissue damage, and, in the end, tooth loss.

This study has limitations, even though the results are promising. The limited sample size and brief follow-up necessitate cautious interpretation of the findings. Not every instance treated with lasers got the best results, which could be due to the severity of the lesion or the response of the host. The single-center design and precise laser settings may restrict generalizability; there is a necessity for protocol standardization and replication. Subsequent research ought to differentiate the effects of PBM from disinfection and integrate biological markers to elucidate processes. In conclusion, our preliminary data suggest that a meticulously phased diode laser technique can reconcile endodontic and periodontal healing by cleaning the area and actively promoting repair—aligning with minimally invasive, regenerative methodologies in dentistry. By utilizing the combined antibacterial and photobiomodulatory characteristics of diode lasers, doctors may obtain results in endo-perio lesions that are challenging to achieve through traditional procedures alone [75]; the notion of “laser-guided endo-perio regeneration” necessitates more extensive, long-term studies.

## 6. Brief Literature Review

Background & complexity. Combined endodontic–periodontal lesions are recognized as diagnostically challenging, bidirectional infections that need coordinated endo–perio care and staged decision-making [68]. Why diode lasers/PBM? Diode lasers (≈810–980 nm) offer photothermal decontamination and photobiomodulation (PBM) that modulates inflammation and supports healing; mechanistic and clinical reviews support these dual actions.

Periodontal outcomes (adjunct to SRP). Recent trials and syntheses show modest, protocol-dependent improvements in PD/BOP—and occasional CAL gains—when diode lasers are added to non-surgical therapy; effects are heterogeneous and depend on wavelength, power, and timing [64,76,77,78].

Endodontic pain control. Multiple clinical studies report lower early postoperative pain when PBM/diode application accompanies RCT, especially at 24–48 h [34,35,79].

Disinfection signal. Contemporary reviews indicate diode-assisted (and related laser) protocols can reduce intracanal bacterial load, though results are parameter-sensitive and standardization are urged [79,80,81].

Antibiotic-sparing context. Post-2015 guidance limits systemic antibiotics in endodontics to cases with systemic involvement, supporting localized adjuncts like lasers when appropriate [72,82,83].

The gap our study addresses. Most published uses (Table 2) are single-stage (disinfection or PBM); there is a need to test sequential, phase-matched protocols aligned with infection control → inflammation modulation → regeneration—precisely the rationale of your multi-stage approach [84].

## 7. Limitations and Future Directions

This study has several limitations:

Small sample size and non-randomized design: The case series nature limits strong inference and generalizability.

Short follow-up (6 months): Periodontal and bone regeneration often continue beyond 6 months; longer-term data are needed to confirm stability.

Single-center operator bias: All treatments were done by the same clinician; multicenter replication would strengthen external validity.

Lack of biological markers: We did not collect inflammatory cytokine or growth factor data to mechanistically validate the photobiomodulation effects.

Future research should include randomized controlled trials with larger sample sizes, blinded evaluators, and longer follow-ups (6–12 months). Inclusion of biomarker analyses (e.g., cytokines, collagen synthesis, angiogenic markers) would help validate the mechanistic basis. A third arm using only a disinfection laser (without photobiomodulation phases) could isolate the relative contributions of each phase.

If validated, this multi-stage protocol could become a clinically adoptable standard, improving predictability in challenging endo-perio cases and reducing reliance on systemic antibiotics.

## 8. Conclusions

This case series indicates that the multi-stage diode laser protocol is feasible, well-tolerated, and safe within the conditions of this study. The observed clinical and radiographic improvements should be interpreted as preliminary observations. Larger, controlled studies are required to determine efficacy and long-term outcomes. Antibiotics have traditionally been administered in the management of endo-perio lesions to mitigate the microbial aspect of infection [87], especially in instances characterized by acute symptoms, systemic involvement, or spreading cellulitis. However, modern guidelines stress a more cautious and evidence-based way to use antibiotics. Current guidelines promote local debridement and definitive endodontic-periodontal therapy as the major methods for infection prevention, with systemic antibiotics reserved for certain purposes [88]. This change is due to worries about antibiotic resistance, negative effects on the whole body, and the fact that most localized infections can be treated well with mechanical and chemical methods. In this situation, laser-assisted disinfection is a good option since it targets the microbial biofilm locally with little damage to other areas, lowers the bacterial load beyond the reach of standard instruments, and may even make systemic antibiotics unnecessary in many cases. As a result, adding laser therapy to the mix is in line with the concepts of antimicrobial stewardship, which has both therapeutic and public health benefits by reducing unnecessary antibiotic exposure [89]. Antibiotics are often ineffective in avascular intracanal environments, and their overuse contributes to resistance [90].

These preliminary findings suggest that photobiomodulation may help support tissue healing, promote bone regeneration, and enhance patient comfort in complex endodontic-periodontal cases. This method may also lessen the need for systemic antibiotics and support a more biologically driven, minimally invasive approach to treatment. These results are particularly promising when considered alongside our previous findings demonstrating the dual capacity of laser radiation for both microbial decontamination and photobiomodulation-mediated healing, aspects that have been extensively investigated by our research group [91,92,93,94].

However, these results should be viewed as early evidence rather than proof of clinical efficacy. Larger, multicenter, controlled trials with longer follow-up are needed to confirm these outcomes. Future studies exploring molecular and histological markers could also help clarify how photobiomodulation contributes to regeneration. If confirmed, this multi-stage laser protocol could become a valuable addition to the clinical management of endodontic-periodontal lesions.

## Figures and Tables

**Table 1:** Baseline vs. 6-Month Outcomes—Descriptive Statistics, Confidence Intervals, Effect Sizes, and Wilcoxon Tests. Values represent paired baseline vs. 6-month outcomes (*n* = 12). Means are shown as Mean ± SD with 95% confidence intervals derived from 10,000-resample bootstrap estimates. The Wilcoxon signed-rank test was two-tailed (α = 0.05). Negative changes indicate reductions (improvement) for probing depth (PD) and bleeding on probing (BOP). Both confidence intervals and effect sizes are included to facilitate interpretation given the exploratory nature and limited sample size of this pilot study.

Parameter	Baseline	6 Months	Median Change (IQR)
Probing depth (mm)	7.6 (7.0–8.0)	6.0 (5.5–6.5)	−1.5 (−2.0 to −1.0)
Bleeding on probing (proportion)	0.9 (0.8–1.0)	0.3 (0.0–0.5)	−0.6 (−0.8 to −0.4)
Bone fill (%)	—	58.3 (50.0–66.7)	+58.3 (50.0–66.7)
VAS pain score (0–10)	moderate–severe	mild at 24 h	within-patient reduction only (non-comparative)

**Table 2:** Summary of recent (2015−2024) literature on diode-laser/photobiomodulation in endodontic-periodontal therapy: Overview of clinical trials, systematic reviews, and guidelines evaluating diode lasers (≈810–980 nm) as adjuncts to non-surgical periodontal therapy and/or endodontic treatment. Columns list title/authors, year, laser use, protocol/parameters, evaluation metrics, and key results. Across studies, adjunctive lasers show modest, protocol-dependent improvements in PPD/BOP (with occasional CAL gains), consistent early analgesic effects after RCT, and parameter-sensitive disinfection; guidelines remain cautious due to heterogeneity.

Title	Year	Laser Use	Protocol (Design/Params)	Evaluation	Key Results
Treatment of stage I–III periodontitis—The EFP S3 level clinical practice guideline [68]	2020	Various adjunct lasers	Evidence-based CPG synthesis	PPD, CAL, BOP, patient outcomes	Insufficient evidence to recommend adjunct lasers routinely in non-surgical therapy; effects protocol-dependent.
Effectiveness of an 810-nm Diode Laser in Addition to Non-surgical Periodontal Therapy in Patients with Chronic Periodontitis: A Randomized Single-Blind Clinical Trial [76]	2021	810 nm diode (adjunct to SRP)	RCT; SRP±diode laser; short-term follow-up	PPD, CAL, PI, BOP	Modest adjunctive gains vs SRP alone; improvements small and time-dependent.
Evaluation of 940-nm diode laser effectiveness on pocket depth, clinical attachment level, and bleeding on probing in chronic periodontitis: A randomized clinical study [64]	2023	940 nm diode (adjunct)	Parallel RCT; SRP ± 940 nm	PPD, CAL, BOP	Adjunct benefit reported on select parameters at early follow-up; heterogeneity noted.
Evaluating the Effects of Diode Laser 940 nm Adjunctive to Conventional Scaling and Root Planning for Gingival Sulcus Disinfection in Chronic Periodontitis Patients [85]	2024	940 nm diode (adjunct)	Split-mouth RCT; 4–8 wk	PPD, CAL, GI, BOP	Limited additional benefit overall; CAL gains at 4 & 8 wk; PD/BOP not significantly different.
Clinical efficacy and pain control of diode laser-assisted flap surgery in the treatment of chronic periodontitis: A systematic review and meta-analysis [78]	2024	Diode (810–980 nm)	Meta-analysis of RCTs	PPD (3–6 mo), pain	PPD reduction at 3 & 6 mo; analgesic benefit indicated; effect sizes vary by protocol.
Effect of a 980-nm diode laser on post-operative pain after endodontic treatment in teeth with apical periodontitis: A randomized clinical trial [34]	2021	980 nm diode (post-RCT)	RCT; laser after chemo-mechanical prep	VAS pain at 6–48 h	Significantly lower pain at early time points vs control.
Efficiency of diode laser in control of post-endodontic pain: A randomized controlled trial [35]	2023	810/980 nm (LLLT vs. LAI vs. manual)	3-arm RCT (LLLT, LAI, manual)	VAS pain 24–72 h	LLLT best at 24 h; at 48 h LLLT ≈ LAI, both < manual; differences fade by 72 h.
Effect of photobiomodulation on postoperative endodontic pain: A systematic review of clinical trials [80]	2024	PBM (810–980 nm)	Systematic review of clinical trials	Post-endo pain	7/9 studies showed significant pain reduction with PBM vs. controls.
The efficacy of 2780 nm Er, Cr: YSGG and 940 nm Diode Laser in root canal disinfection: A randomized clinical trial [81]	2024	940 nm diode (intracanal)	Narrative/structured review	Bacterial load, disinfection	Significant bacterial reduction with laser-assisted disinfection; parameter-dependence emphasized.
Efficacy of diode laser (980 nm) and non-surgical therapy on management of periodontitis: A randomized clinical trial [86]	2021	980 nm diode (adjunct)	Split-mouth RCT; single-dose 980 nm + SRP vs. SRP	PPD, PI, bleeding indices	Greater reductions in PPD and bleeding vs. SRP alone over 9 mo; microbiological reduction reported.

## Data Availability

The data will be available from the corresponding authors upon reasonable requests and respecting privacy and ethical restrictions.

## Abbreviations

The following abbreviations are used in this manuscript:

Abbreviation	Definition
PD	Probing depth
CAL	Clinical attachment level
BOP	Bleeding on probing
VAS	Visual analog scale (0–10)
PBM	Photobiomodulation
LLLT	Low-level laser therapy (synonym of PBM)
LAI	Laser-activated irrigation
SRP	Scaling and root planing
RCT (treatment)	Root canal treatment
RCT (trial design)	Randomized controlled trial
ROI	Region of interest (for image analysis)
CEJ	Cemento-enamel junction
CBCT	Cone-beam computed tomography
Rx bone fill (%)	Radiographic bone fill (percentage)

## References

[1] Rotstein I., Simon J.H. (2004). Diagnosis, prognosis and decision-making in the treatment of combined periodontal–endodontic lesions. Periodontol. 2000.

[2] Chen B., Zhu Y., Lin M., Zhang Y., Li Y., Ouyang X., Ge S., Lin J., Pan Y., Xu Y. (2024). Expert consensus on the diagnosis and therapy of endo-periodontal lesions. Int. J. Oral Sci..

[3] Al-Fouzan K.S. (2014). A new classification of endodontic-periodontal lesions. Int. J. Dent..

[4] Siqueira J.F., Rôças I.N. (2022). Present status and future directions: Microbiology of endodontic infections. Int. Endod. J..

[5] Nair P.N. (2004). Pathogenesis of apical periodontitis and the causes of endodontic failures. Crit. Rev. Oral Biol. Med..

[6] Kandaswamy D., Venkateshbabu N. (2010). Root canal irrigants. J. Conserv. Dent..

[7] Kim S., Kratchman S. (2006). Modern endodontic surgery concepts and practice: A review. J. Endod..

[8] Herrera D., van Winkelhoff A.J., Matesanz P., Lauwens K., Teughels W. (2023). Europe’s contribution to the evaluation of systemic antimicrobials in the treatment of periodontitis. Periodontol. 2000.

[9] Herrera D., Matesanz P., Martín C., Oud V., Feres M., Teughels W. (2020). Adjunctive effect of locally delivered antimicrobials in periodontitis therapy: A systematic review and meta-analysis. J. Clin. Periodontol..

[10] Al-Sibassi A., Niazi S., Clarke P., Adeyemi A. (2025). Management of the endodontic-periodontal lesion. Br. Dent. J..

[11] Ricucci D., Siqueira J.F. (2010). Biofilms and apical periodontitis: Study of prevalence and association with clinical and histopathologic findings. J. Endod..

[12] El-Kishawi M., Khalaf K. (2021). An update on root canal preparation techniques and how to avoid procedural errors in endodontics. Open Dent. J..

[13] Santos M.S., Silva J.C., Carvalho M.S. (2024). Hierarchical biomaterial scaffolds for periodontal tissue engineering: Recent progress and current challenges. Int. J. Mol. Sci..

[14] Cortellini P., Tonetti M.S. (2015). Clinical concepts for regenerative therapy in intrabony defects. Periodontol. 2000.

[15] Trentin M.S., Dallepiane F.G., Figueiredo P.S., Becker A.L., Leocovick S., Duque T.M., De Carli J.P., Vanni J.R. (2024). Management of Endo-Perio Lesion in a Tooth with an Unfavorable Prognosis: A Clinical Case Report with an 18-Month Follow-Up. Odovtos.

[16] Maheswary T., Nurul A.A., Fauzi M.B. (2021). The insights of microbes’ roles in wound healing: A comprehensive review. Pharmaceutics.

[17] Verma P.K., Srivastava R., Gupta K.K., Srivastava A. (2011). Combined endodontic-periodontal lesion: A clinical dilemma. J. Interdiscip. Dent..

[18] Vieira A., Siqueira J.F., Lopes W.S.P. (2012). Dentinal tubule infection as the cause of recurrent disease and late endodontic treatment failure: A case report. J. Endod..

[19] Wong J., Manoil D., Näsman P., Belibasakis G.N., Neelakantan P. (2021). Microbiological aspects of root canal infections and disinfection strategies: An update review. Front. Oral Health.

[20] Keinan D., Nuni E., Bronstein Rainus M., Ben Simhon T., Dakar A., Slutzky-Goldberg I., Dakar R. (2024). Retreatment of failed regenerative endodontic therapy: Outcome and treatment considerations. Cureus.

[21] Tavanafar S., Karimpour A., Karimpour H., Saleh A.M., Saeed M.H. (2015). Effect of different instrumentation techniques on vertical root fracture resistance of endodontically treated teeth. J. Dent..

[22] Gnanadhas D., Elango M., Janardhanraj S., Srinandan C.S., Datey A., Strugnell R.A., Gopalan J., Chakravortty D. (2015). Successful treatment of biofilm infections using shock waves combined with antibiotic therapy. Sci. Rep..

[23] Pérez A.R., Rendón J., Ortolani-Seltenerich P.S., Pérez-Ron Y., Cardoso M., Noites R., Loroño G., Vieira G.C.S. (2025). Extraradicular Infection and Apical Mineralized Biofilm: A Systematic Review of Published Case Reports. J. Clin. Med..

[24] Zhao M., Xie Y., Gao W., Li C., Ye Q., Li Y. (2023). Diabetes mellitus promotes susceptibility to periodontitis—Novel insight into the molecular mechanisms. Front. Endocrinol..

[25] Aranda Verdú S., Pallarés Sabater A., Pallarés Serrano A., Rubio Climent J., Casino Alegre A. (2025). Endo-periodontal lesions without root damage: Recommendations for clinical management. J. Clin. Med..

[26] Wu H., Li Y., Shi L., Liu Y., Shen J. (2025). New advances in periodontal functional materials based on antibacterial, anti-inflammatory, and tissue regeneration strategies. Adv. Healthc. Mater..

[27] Sachelarie L., Cristea R., Burlui E., Hurjui L.L. (2024). Laser technology in dentistry: From clinical applications to future innovations. Dent. J..

[28] Khadhim Ghafla A., Aalipour R., Khlaif H., Sarhan S. (2024). The therapeutic efficacy of different power of diode laser in surgically apicectomized teeth. Nanotechnol. Percept..

[29] Asnaashari M., Sadeghian A., Hazrati P. (2022). The effect of high-power lasers on root canal disinfection: A systematic review. J. Lasers Med. Sci..

[30] Gutknecht N., Gogswaardt D., Conrads G., Apel C., Schubert C., Lampert F. (2000). Diode laser radiation and its bactericidal effect in root canal wall dentin. J. Clin. Laser Med. Surg..

[31] Wenzler J.S., Falk W., Frankenberger R., Braun A. (2021). Impact of adjunctive laser irradiation on the bacterial load of dental root canals: A randomized controlled clinical trial. Antibiotics.

[32] Ayed Y., Al-Haddad A., Kassab A., Alhodhodi A., Dar-Odeh N., Ragheb Y.S., Elbaghir S.M., Elsayed S.A. (2025). From Biological Mechanisms to Clinical Applications: A Review of Photobiomodulation in Dental Practice. Photobiomodul. Photomed. Laser Surg..

[33] Dembowska E., Jaroń A., Homik-Rodzińska A., Gabrysz-Trybek E., Bladowska J., Trybek G. (2022). Comparison of the treatment efficacy of endo-perio lesions using a standard treatment protocol and extended by using a diode laser (940 nm). J. Clin. Med..

[34] Kaplan T., Sezgin G.P., Sönmez Kaplan S. (2021). Effect of a 980-nm diode laser on post-operative pain after endodontic treatment in teeth with apical periodontitis: A randomized clinical trial. BMC Oral Health.

[35] Ismail H.H., Obeid M., Hassanien E. (2023). Efficiency of diode laser in control of post-endodontic pain: A randomized controlled trial. Clin. Oral Investig..

[36] Machado G.G.A., Ferreira G.F., Mello E.S., Ando-Suguimoto E.S., Roncolato V.L., Oliveira M.R.C., Tognini J.A., Paisano A.F., Camacho C.P., Bussadori S.K. (2025). Effect of photobiomodulation on post-endodontic pain following single-visit treatment: A randomized double-blind clinical trial. J. Pers. Med..

[37] Guimarães L.D., da Silva E.A., Hespanhol F.G., Fontes K.B.F.d.C., Antunes L.A.A., Antunes L.S., Silva E., Antunes L. (2021). Effect of photobiomodulation on post-operative symptoms in teeth with asymptomatic apical periodontitis treated with foraminal enlargement: A randomized clinical trial. Int. Endod. J..

[38] World Medical Association (2013). World Medical Association Declaration of Helsinki: Ethical principles for medical research involving human subjects. JAMA.

[39] Bartols A., Bormann C., Werner L., Schienle M., Walther W., Dörfer C.E. (2020). A retrospective assessment of different endodontic treatment protocols. PeerJ.

[40] Peters O.A., Rossi-Fedele G., George R., Kumar K., Timmerman A., Wright P.P. (2024). Guidelines for non-surgical root canal treatment. Aust. Endod. J..

[41] Srivastava S. (2024). Root canal instrumentation: Current trends and future perspectives. Cureus.

[42] Petrovski M., Veljanovski D., Nikolovski B., Mladenovski M., Kovacevska I. (2024). Contemporary aspects of treatment of endodontal–periodontal lesions. Mathews J. Dent..

[43] Herrera D. (2016). Scaling and root planing is recommended in the nonsurgical treatment of chronic periodontitis. J. Evid. Based Dent. Pract..

[44] Plessas A. (2014). Nonsurgical periodontal treatment: Review of the evidence. Oral Health Dent. Manag..

[45] Marcattili D., Mancini L., Tarallo F., Casalena F., Pietropaoli C., Marchetti E. (2023). Efficacy of two diode lasers in the removal of calculus from the root surface: An in vitro study. Clin. Exp. Dent. Res..

[46] Parker S., Cronshaw M. (2025). Laser safety in dental practice in the United Kingdom. Br. Dent. J..

[47] Kivanç B.H., Arisu H.D., Saglam B.C., Akca G.Ü., Gurel M.A., Gorgul G. (2017). Evaluation of antimicrobial and thermal effects of diode laser on root canal dentin. Niger. J. Clin. Pract..

[48] Dompe C., Moncrieff L., Matys J., Grzech-Leśniak K., Kocherova I., Bryja A., Bruska M., Dominiak M., Mozdziak P., Skiba T.H.I. (2020). Photobiomodulation—Underlying mechanism and clinical applications. J. Clin. Med..

[49] Pawar S., Pujar M., Makandar S., Khaiser M. (2014). Postendodontic treatment pain management with low-level laser therapy. J. Dent. Lasers..

[50] Salceanu M., Melian A., Hamburda T., Antohi C., Concita C., Topoliceanu C., Giuroiu C.L. (2025). Imaging techniques in endodontic diagnosis: A review of literature. Rom. J. Oral Rehabil..

[51] Ali M., Irfan M., Ali T., Wei C.R., Akilimali A. (2025). Artificial intelligence in dental radiology: A narrative review. Ann. Med. Surg..

[52] Fitzgerald B.P., Hawley C.E., Harrold C.Q., Garrett J.S., Polson A.M. (2022). Reproducibility of manual periodontal probing following a comprehensive standardization and calibration training program. J. Oral Biol..

[53] Stødle I.H., Imber C., Shanbhag S.V., Salvi G.E., Verket A., Stähli A. (2025). Methods for clinical assessment in periodontal diagnostics: A systematic review. J. Clin. Periodontol..

[54] Armitage G.C. (1999). Development of a classification system for periodontal diseases and conditions. Ann. Periodontol..

[55] Simon J.H., Glick D.H., Frank A.L. (1972). The relationship of endodontic-periodontic lesions. J. Periodontol..

[56] Kim G.Y., Kim S., Chang J.S., Pyo S.W. (2023). Advancements in methods of classification and measurement used to assess tooth mobility: A narrative review. J. Clin. Med..

[57] Nathani T.I., Olivieri J.G., Tomás J., Elmsmari F., Abella F., Durán-Sindreu F. (2024). Post-operative pain after single-visit root canal treatment using resin-based and bioceramic sealers in teeth with apical periodontitis: A randomised controlled trial. Aust. Endod. J..

[58] Jundaeng J., Chamchong R., Nithikathkul C. (2025). Advanced AI-assisted panoramic radiograph analysis for periodontal prognostication and alveolar bone loss detection. Front. Dent. Med..

[59] Kui A., Paraschiv A.M., Pripon M., Chisnoiu A.M., Iacob S., Berar A., Popa F., Gorcea S., Buduru S. (2025). From Pain to Recovery: The Impact of Laser-Assisted Therapy in Dentistry and Cranio-Facial Medicine. Balneo PRM Res. J..

[60] Khattab S.F., Gomaa Y.F., Abdelaziz E.A.E., Khattab N.M.A. (2025). Influence of photobiomodulation therapy on regenerative potential of non-vital mature permanent teeth in healthy canine dogs. Eur. Arch. Paediatr. Dent..

[61] Al Asmari D., Alenezi A. (2025). Laser technology in periodontal treatment: Benefits, risks, and future directions—A mini review. J. Clin. Med..

[62] Frankowski D.W., Ferrucci L., Arany P.R., Bowers D., Eells J.T., Gonzalez-Lima F., Lohr N.L., Quirk B.J., Whelan H.T., Lakatta E.G. (2025). Light buckets and laser beams: Mechanisms and applications of photobiomodulation therapy. GeroScience.

[63] Lee S.B., Lee H., Park J.B. (2025). Low-level laser therapy enhances osteogenic differentiation of gingiva-derived stem cells in 2D and 3D cultures. Sci. Rep..

[64] Seyed-Monir A., Seyed-Monir E., Mihandoust S. (2023). Evaluation of 940-nm diode laser effectiveness on pocket depth, clinical attachment level, and bleeding on probing in chronic periodontitis: A randomized clinical study. Dent. Res. J..

[65] Ren C., McGrath C., Jin L., Zhang C., Yang Y. (2017). The effectiveness of low-level laser therapy as an adjunct to non-surgical periodontal treatment: A meta-analysis. J. Periodontal Res..

[66] Parker S., Cronshaw M., Anagnostaki E., Mylona V., Lynch E., Grootveld M. (2020). Current Concepts of Laser–Oral Tissue Interaction. Dent. J..

[67] Hasturk H., Kantarci A. (2015). Activation and resolution of periodontal inflammation and its systemic impact. Periodontol. 2000.

[68] Sanz M., Herrera D., Kebschull M., Chapple I., Jepsen S., Beglundh T., Sculean A., Tonetti M.S., EFP Workshop Participants and Methodological Consultants (2020). Treatment of stage I–III periodontitis—The EFP S3 level clinical practice guideline. J. Clin. Periodontol..

[69] Berni M., Brancato A.M., Torriani C., Bina V., Annunziata S., Cornella E., Trucchi M., Jannelli E., Mosconi M., Gastaldi G. (2023). The role of low-level laser therapy in bone healing: Systematic review. Int. J. Mol. Sci..

[70] Pedraza F.H., Teves-Cordova A.V.I., Alcalde M.P., Duarte M.A.H. (2025). Impact of the use of high-power 810-nm diode laser as monotherapy on the treatment of teeth with periapical lesions. Restor. Dent. Endod..

[71] Hazrati P., Azadi A., Tizno A., Asnaashari M. (2024). The effect of lasers on the healing of periapical lesion: A systematic review. J. Lasers Med. Sci..

[72] Amaroli A., Colombo E., Zekiy A., Aicardi S., Benedicenti S., De Angelis N. (2020). Interaction between laser light and osteoblasts: Photobiomodulation as a trend in the management of socket bone preservation—A review. Biology.

[73] Boddun M., Sharva V. (2020). Laser hazards and safety in dental practice: A review. Oral Health Care.

[74] Hoshyari N., Mesgarani A., Sheikhi M.M., Goli H., Nataj A.H., Chiniforush N. (2024). Comparison of antimicrobial effects of 445 and 970 nm diode laser irradiation with photodynamic therapy and triple antibiotic paste on *Enterococcus faecalis* in the root canal: An in vitro study. Maedica.

[75] Arany P.R. (2024). Photobiomodulation Therapy. JADA Found. Sci..

[76] Mokhtari M.R., Ahrari F., Dokouhaki S., Fallahrastegar A., Ghasemzadeh A. (2021). Effectiveness of an 810-nm Diode Laser in Addition to Non-surgical Periodontal Therapy in Patients with Chronic Periodontitis: A Randomized Single-Blind Clinical Trial. J. Lasers Med. Sci..

[77] Aggarwal R., Bawa S.S., Palwankar P., Kaur S., Choudhary D., Kochar D. (2023). To Evaluate the Clinical Efficacy of 940 nm Diode Laser and Propolis Gel (A Natural Product) in Adjunct to Scaling and Root Planing in Treatment of Chronic Periodontitis. J. Pharm. Bioallied Sci..

[78] Hu Q., Liu X., Zhao Z., Guo Z., Liu Q., Liu N. (2024). Clinical efficacy and pain control of diode laser-assisted flap surgery in the treatment of chronic periodontitis: A systematic review and meta-analysis. Heliyon.

[79] Bansode S.H., Chole D.G., Bakle S.S., Hatte N.R., Gandhi N.P., Inamdar M.R. (2025). In vivo evaluation of root canal disinfection using a combination of ultrasonic activation and diode laser therapy. J. Conserv. Dent. Endod..

[80] Seyyedi S.A., Fini M.B., Fekrazad R., Abbasian S., Abdollahi A.A. (2024). Effect of photobiomodulation on postoperative endodontic pain: A systematic review of clinical trials. Dent. Res. J..

[81] Fahim S.Z., Ghali R.M., Hashem A.A., Farid M.M. (2024). The efficacy of 2780 nm Er,Cr:YSGG and 940 nm Diode Laser in root canal disinfection: A randomized clinical trial. Clin. Oral Investig..

[82] Galić M., Miletić I., Poklepović Peričić T., Rajić V., Jurčević N.N.V., Pribisalić A., Mikić I.M. (2024). Antibiotic Prescribing Habits in Endodontics among Dentists in the Federation of Bosnia and Herzegovina—A Questionnaire-Based Study. Antibiotics.

[83] Winkler P.C., Benz L., Nickles K., Petsos H.C., Eickholz P., Dannewitz B. (2024). Decision-making on systemic antibiotics in the management of periodontitis: A retrospective comparison of two concepts. J. Clin. Periodontol..

[84] Martínez-García M., Hernández-Lemus E. (2021). Periodontal inflammation and systemic diseases: An overview. Front. Physiol..

[85] Poormoradi B., Rabinezhad N., Mohamadpour L., Kazemi M., Farhadian M. (2024). Evaluating the Effects of Diode Laser 940 nm Adjunctive to Conventional Scaling and Root Planning for Gingival Sulcus Disinfection in Chronic Periodontitis Patients. Avicenna J. Dent. Res..

[86] Faragalla A., Awooda E., Bolad A., Ghandour I. (2021). Efficacy of diode laser (980 nm) and non-surgical therapy on management of periodontitis: A randomized clinical trial. J. Res. Med. Dent. Sci..

[87] Yadav D., Abhilash A., Mani E.S., Hari K., Arya A. (2025). Clinical efficacy of locally delivered antibiotics in treating endodontic-periodontal lesions. J. Pharm. Bioallied Sci..

[88] Amato M., Santonocito S., Polizzi A., Tartaglia G.M., Ronsivalle V., Viglianisi G., Grippaudo C., Isola G. (2023). Local delivery and controlled release drug systems: A new approach for the clinical treatment of periodontitis therapy. Pharmaceutics.

[89] Kaiwusaer A. (2025). Laser-assisted biofilm disruption and its role in periodontal tissue regeneration. Theor. Nat. Sci..

[90] AAE Position Statement (2017). Guidance on the use of systemic antibiotics in endodontics. J. Endod..

[91] Munteanu I.R., Luca R.E., Mateas M., Darawsha L.D., Boia S., Boia E.-R., Todea C.D. (2022). The Efficiency of Photodynamic Therapy in the Bacterial Decontamination of Periodontal Pockets and Its Impact on the Patient. Diagnostics.

[92] Munteanu I.-R., Luca R.-E., Hogea E., Erdelyi R.-A., Duma V.-F., Marsavina L., Globasu A.-L., Constantin G.-D., Todea D.C. (2024). Microbiological and Imaging-Based Evaluations of Photodynamic Therapy Combined with Er:YAG Laser Therapy in the In Vitro Decontamination of Titanium and Zirconia Surfaces. Microorganisms.

[93] Luca R.-E., Del Vecchio A., Munteanu I.-R., Margan M.-M., Todea C.D. (2025). Evaluation of the Effects of Photobiomodulation on Bone Density After Placing Dental Implants: A Pilot Study Using Cone Beam CT Analysis. Clin. Pract..

[94] Luca R.E., Giuliani A., Mănescu A., Heredea R., Hoinoiu B., Constantin G.D., Duma V.-F., Todea C.D. (2020). Osteogenic Potential of Bovine Bone Graft in Combination with Laser Photobiomodulation: An Ex Vivo Demonstrative Study in Wistar Rats by Cross-Linked Studies Based on Synchrotron Microtomography and Histology. Int. J. Mol. Sci..

